# The Probiotic Strain *H. alvei* HA4597^®^ Improves Weight Loss in Overweight Subjects under Moderate Hypocaloric Diet: A Proof-of-Concept, Multicenter Randomized, Double-Blind Placebo-Controlled Study

**DOI:** 10.3390/nu13061902

**Published:** 2021-06-01

**Authors:** Pierre Déchelotte, Jonathan Breton, Clémentine Trotin-Picolo, Barbara Grube, Constantin Erlenbeck, Gordana Bothe, Sergueï O. Fetissov, Grégory Lambert

**Affiliations:** 1 Inserm UMR 1073, 76000 Rouen, France; jonathan.breton.pro@gmail.com; 2 Nutrition Department, University Hospital, 76000 Rouen, France; 3 Department of Biology, Rouen Normandy University, 76130 Mont-Saint-Aignan, France; serguei.fetissov@univ-rouen.fr; 4 TargEDys SA, 91160 Longjumeau, France; cpicolo@targedys.com (C.T.-P.); glambert@targedys.com (G.L.); 5 Practice for General Medicine, 12169 Berlin, Germany; praxis@hausarzt-grube-krieger.de; 6 Analyze & Realize GmbH, 13467 Berlin, Germany; erlenbeck@gmx.de (C.E.); GBothe@a-r.com (G.B.); 7 Inserm UMR 1239, 76130 Mont-Saint-Aignan, France

**Keywords:** overweight subjects, gut microbiota, probiotics, *H. alvei* HA4597, HA4597^®^, feeling of fullness, appetite, hip circumference

## Abstract

*Background*: Increasing evidence supports the role of the gut microbiota in the control of body weight and feeding behavior. Moreover, recent studies have reported that the probiotic strain *Hafnia alvei* HA4597^®^ (HA), which produces the satietogenic peptide ClpB mimicking the effect of alpha-MSH, reduced weight gain and adiposity in rodent models of obesity. *Methods*: To investigate the clinical efficacy of HA, 236 overweight subjects were included, after written informed consent, in a 12-week prospective, double-blind, randomized study. All subjects received standardized counselling for a −20% hypocaloric diet and were asked to maintain their usual physical activity. Subjects of the HA group received two capsules per day providing 100 billion bacteria per day and subjects in the Placebo (P) group received two placebo capsules. The primary endpoint was the percentage of subjects achieving a weight loss of at least 3% after 12 weeks. Intention-to-treat statistical analysis was performed using exact-Fischer, Mann-Whitney and paired-Wilcoxon tests as appropriate. *Results*: In the HA group, significantly more subjects (+33%) met the primary endpoint than in the P group (54.9 vs. 41.4%, *p* = 0.048). In the HA group, an increased feeling of fullness (*p* = 0.009) and a greater loss of hip circumference (*p* < 0.001) at 12 weeks were also observed. Fasting glycemia at 12 weeks was significantly lower (*p* < 0.05) in the HA compared to P group. Clinical and biological tolerance was good in both groups. *Conclusions*: A 12-week treatment with the probiotic strain *H. alvei* HA4597^®^ significantly improves weight loss, feeling of fullness and reduction of hip circumference in overweight subjects following moderate hypocaloric diet. These data support the use of *H. alvei* HA4597^®^ in the global management of excess weight.

## 1. Introduction

Excess weight, defined as overweight and obesity, is a global public health concern, with a rapid increase of prevalence and multiple complications [1]. Thus, efficient therapeutic strategies are needed to limit the progression of weight gain. Multifactorial approaches have been proposed, based mainly on dietetic and behavioral changes [2], while pharmacotherapy for obesity or binge eating disorder remains of limited efficacy and poor tolerance for most drugs [3,4]. Over the last decade, intensive effort in gut microbiota’s research allowed to elucidate distinct bacterial signaling pathways related to host energy harvesting, fat deposition, inflammation and insulin resistance in obesity [5,6]. Moreover, a role of gut microbiota in the regulation of host appetite and feeding behavior has been demonstrated in the physiological and pathological situation [7,8]. For instance, gut bacteria-derived proteins interact with host satiety signaling via stimulating the release of intestinal hormones such as glucagon-like peptide 1 (GLP-1) and peptide YY (PYY), and may also activate anorexigenic pathways in hypothalamic and brainstem nuclei [9]. It appears hence interesting to develop new therapeutic strategies for body weight management targeting gut microbial influence on appetite and/or satiety to prevent overeating and progressive overweight and obesity [10]. Therefore, the use of probiotics to achieve anti-obesity effects has been proposed. The potential mechanisms of action of classic probiotics involve the reset of the consequences of gut microbial dysregulations, including reduction of fat storage, promotion of fatty acid oxidation and reduction of low-grade inflammation [11,12,13]. A new way of preventing excess weight gain may rely on the activation of the anorexigenic pathways [8,9,10]. We previously reported that increased production of ClpB by commensal bacteria was associated with increased anorexigenic effects of bacterial proteins administered in normal rats [9]. To achieve such effects in humans, the probiotic strain *Hafnia alvei* HA4597^®^(HA) currently seems a promising candidate. Indeed, this new generation precision probiotic produces the caseinolytic protease B (ClpB) protein, identified as a conformational mimetic of the anorexigenic α-melanocyte-stimulating hormone (α-MSH) [6,8,9,14]. Treatment with HA of obese high-fat-diet (HFD)-fed and leptin-deficient ob/ob hyperphagic mice decreased their body weight gain, fat-mass gain and reduced food intake [15,16]. These effects were associated with reduced hyperglycemia, plasma total cholesterol and alanine aminotransferase, suggesting also an improvement in the metabolic consequences of these obesogenic conditions. Moreover, bacterial ClpB directly activates PYY secretion in the intestinal mucosa and the hypothalamic anorexigenic neurons [9,17]. Thus, early intervention in overweight subjects with this probiotic strain may represent an interesting opportunity to limit the evolution of excess weight gain and offer new perspectives for body weight management. Based on this strong preclinical background, the present study investigated the clinical efficacy of HA on weight loss in overweight subjects under moderate hypocaloric diet.

## 2. Materials and Methods

### 2.1. Study Participants

**Figure 1:**
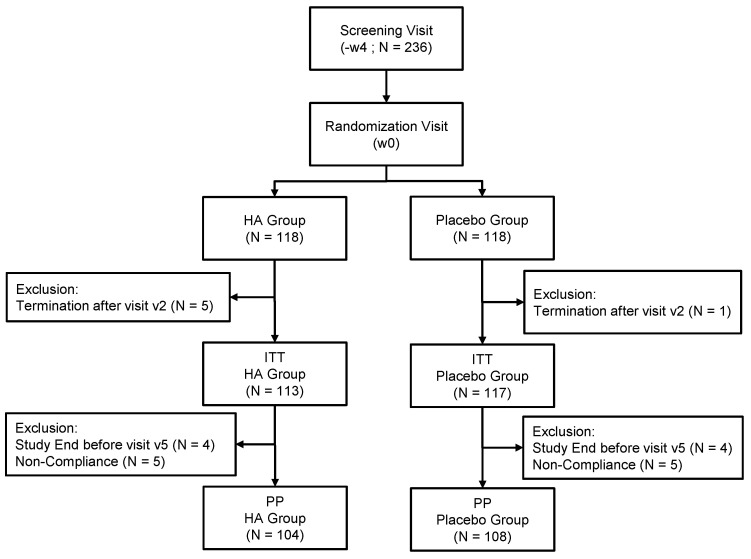
Flowchart illustrating the steps of screening, enrollment, assignment and follow-up of study participants for the Intent To Treat (ITT) and Per Protocol (PP) analysis.

Eligible subjects included into the study were overweight (body mass index, BMI: 25–29.9 kg/m^2^) males and females between 18 and 65 years old, generally in good health with a stable body weight in the last 3 months prior to the study, stable concomitant medications, and, for women of childbearing age, a negative pregnancy test at first study visit as well as commitment to use contraceptive methods. Subjects with known allergy or sensitivity to any component of the investigational product were excluded from the study. Other exclusion criteria included untreated or non-stabilized thyroid gland disorder, hypertension, or type 1 or 2 diabetes, as well as gastrointestinal disorders or gastrointestinal surgery, acute chronic psychotic disorder, immunodeficiency, any other organic or systemic diseases that could influence the conduct and/or outcome of the study, history and/or presence of eating disorders, any electronic medical implant, clinically significant deviations of safety laboratory parameters at first study visit, use of medication or supplementation that could interfere with the study conduct or evaluation or diet/weight loss programs in the last month prior to the first study visit, any restrictive diet such as vegetarian or vegan, pregnancy or nursing, or a history of past or current abuse of drugs, alcohol, medication, participation in another study during the last 30 days prior to enrollment. Smoking itself was not an exclusion criterion. A total of 236 subjects gave written informed consent and were randomly assigned (see flow chart in Figure 1) to “HA” group or “Placebo (P)”, n = 118 for each group. The study protocol was approved by the Ethics Committee of Charité University Berlin and was performed according to the principles of the World Health Organization (Declaration of Helsinki), and of Good Clinical Practice (EMA/CHMP/ICH/135/1995), ICH E6 (R2). The current study has been registered on clinicaltrials.gov (https://clinicaltrials.gov/ct2/show/NCT03657186?term=Hafnia&draw=2& rank=1; (accessed on 4 September 2018)). 

### 2.2. Study Design and Intervention

This placebo-controlled, randomized, double-blind explorative study design was conducted to evaluate the benefits of *Hafnia alvei* HA4597^®^ (HA) on weight reduction in the context of a moderate hypocaloric diet in overweight subjects. An estimation of the sample size has been based on data from previous weight management trials with natural products in addition to dietetic counseling [18,19]. In these studies, the proportion of subjects who lost at least 3% of baseline body weight at 12 weeks (named as “3% responders”) for placebo were ranging roughly from 20% to 30% and for the tested verum products from 50% to 75%. Under the assumption for the present study that about 25% of subjects would have been “3% responders” in the placebo group at least 45% in the verum group, the estimated sample size needed per group was 100 subjects, supposing α = 5% (two-sided) and power of 80%. Taking into account the expected drop-out rate of 15%, a total of 236 subjects were randomized. After screening for inclusion and non-inclusion criteria, the investigational staff provided instructions from trained dietitians to subjects on how to maintain a nutritionally balanced and hypocaloric diet according to individual diet plans throughout the study. The individual energy requirements were calculated based on BMI and reported activity levels at screening (Institute of Medicine, Dietary Reference Intakes for Energy, Carbohydrate, Fiber, Fat, Fatty Acids, Cholesterol, Protein, and Amino Acids (Macronutrients) The National Academy Press, 2005), and energy intake was then reduced by 20%. Subjects were instructed to record their daily intake in diaries and not to increase their assigned energy intake by more than 10%, but to freely decrease it. They were also instructed to maintain their usual physical activity level. Subjects were then randomized (see flowchart in Figure 1) to receive either the investigational study product (IP) containing Hafnia alvei or placebo capsule twice daily, for a total dose of 100 billion bacteria per day in the HA group. The *H. alvei* HA4597^®^ strain is manufactured for TargEDys SA by Biodis (Noyant, France).

### 2.3. Measurements and Endpoints

Clinical parameters including body weight measurements were collected every 4 w until 12 w after randomization and treatment initiation. Body weight (measured using calibrated weighing scales, BC-420MA, Tanita, la Garenne Colombe, FRANCE la, body fat and fat free mass (assessed by bioelectrical impedance analysis, BIA, Tanita Body Composition Analyzer BC-420-MA), BMI (body weight (kg)/(height [m])²), waist and hip circumferences, blood pressure, pulse, laboratory parameters (lipid and carbohydrate metabolism parameters total cholesterol, HDL- and LDL-cholesterol, fasting glucose, and HbA1c), evaluation of the feeling of satiety, fullness, and craving, general well-being parameters (IWQOL-LITE), and global evaluation of benefit of the investigational product (IP) by subject and investigator were also analyzed. The primary endpoint was the proportion of subjects who lost at least 3% of baseline body weight (“3% responders”) at week 12. The secondary endpoints were the feeling of satiety and fullness, the reduction of waist and hip circumferences, the changes in body composition and IWQOL-LITE. The proportion of subjects who lost at least 4% of baseline body weight (“4% responders”) at week 12 was also analyzed as a post-hoc endpoint. 

Safety and tolerability parameters included assessment of adverse events (AE), vital signs (blood pressure, pulse rate), safety laboratory parameters and global evaluation of tolerability of HA or P by subject and investigator. Biological parameters used for safety blood parameters were full blood count parameters (hemoglobin, hematocrit, erythrocytes, thrombocytes, and leucocytes) and liver and renal function parameters (alanine transaminase, aspartate aminotransferase, gamma-GT, alkaline phosphatase, bilirubin, creatinine, urea, and uric acid). Other parameters assessed during the course of the study included stool frequency (assessed in subject diary), global physical activity according to the Global Physical Activity and gastrointestinal symptoms according to the Gastrointestinal Symptom Rating Scale [20], both filled in by the subjects.

### 2.4. Statistical Analysis

All endpoints as well as the concurrent and safety variables received an explorative examination and were descriptively assessed. For metric data (continuous data), the statistical characteristics were given (number, mean, standard deviation, median, extremes, quartiles). For ordinal data (discrete data), number, median, interquartile range and extremes were calculated. For nominal data, the frequency distribution was presented in frequency tables. 

The following exploratory statistical tests were applied:

- Mann-Whitney-U test for comparison of independent groups (u),
- Paired Wilcoxon test for the pre-post comparisons within groups (wil),
- Exact Fisher’s test for the comparison of frequencies for independent groups (exF).

Because of the exploratory character of the study, no adjustment for multiple testing was accomplished. Data were analyzed according to Intention to treat (ITT), Per Protocol (PP) and safety set.

Safety set included all subjects who were randomized and had consumed the investigational product (IP) at least once. In cases where all dispensed IP was returned, the subject was to be considered “non-treated” and was not to be included in the safety set. The ITT set consisted of all subjects in the safety set for whom main benefit parameter (body weight) was available. The PP set consisted of all subjects in the ITT terminating the study without any important deviation of the protocol and its procedures. 

Subjects who had been enrolled in the study according to the inclusion/exclusion criteria, but who were later found to have an important protocol deviation against these criteria at the time point of inclusion in the study (noticed during the study or during the process of data cleaning) were excluded from the ITT, but remained in the Safety population. The protocol deviations were classified as ‘minor’ or ‘major’ in the blinded Data Review Report. Important (‘major’) deviations led to the exclusion of a subject from the PP.

## 3. Results

### 3.1. Subjects

Of 300 subjects assessed for eligibility, 236 subjects were randomized. Of these, six subjects terminated the study immediately after randomization and were excluded from all analysis populations, leaving a set of 230 subjects for the ITT analysis. Six subjects terminated the study after visits at 4w and two others after visit at 8w; nine subjects had major deviations with respect to compliance with IP intake, and one subject had a major deviation regarding compliance with energy intake. Thus, these eighteen subjects were excluded from the ITT population, leaving a set of 212 subjects for the PP analysis. The age of the subjects ranged between 20 and 65 years in HA group and 23 and 65 years in P group. There were no statistical differences in age, gender or other physical/physiological characteristics between the study groups (Table 1).

### 3.2. Effects of the Probiotic strain HA4597^®^

#### 3.2.1. Primary Endpoint

**Figure 2:**
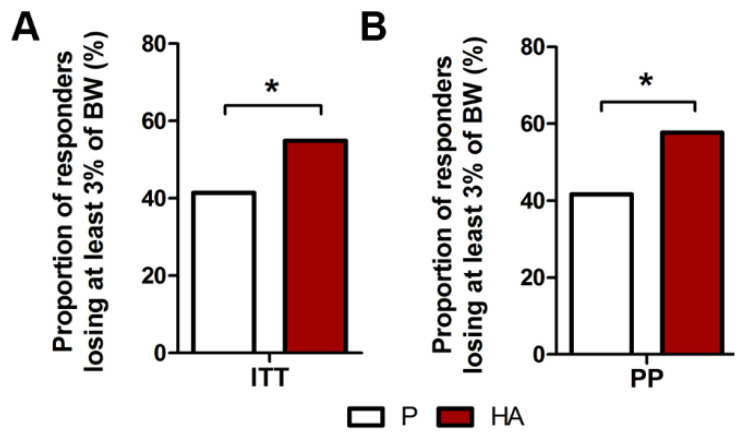
Effect of HA supplementation on the proportion of responders losing at least 3% of body weight. Proportion of overweight subjects losing at least 3% of body weight in ITT (**A**) and PP (**B**) populations. (**A**,**B**) Exact Fisher’s test P. vs. HA.* *p* ≤ 0.05.

In both ITT and PP population, the proportion of subjects who lost at least 3% of baseline body weight was significantly higher in the HA group (54.9% and 57.7%), compared to the placebo group (41.4% and 41.7%, for ITT and PP, respectively) after 12 weeks (*p* = 0.048 and 0.028, respectively), as displayed in Figure 2A,B.

#### 3.2.2. Secondary and Post-Hoc Endpoints 

**Figure 3:**
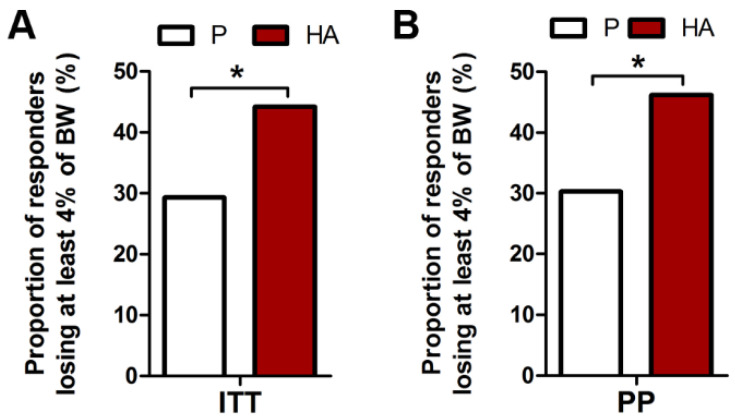
Proportion of responders losing at least 4% of body weight after 12 HA supplementation. Proportion of HA treated subjects losing at least 4% of body weight in ITT (**A**) and PP (**B**) populations. Exact Fisher’s test P. vs. HA.* *p* ≤ 0.05.

Similarly to the primary endpoint, a higher proportion of responders with at least 4% of body weight loss was found, both in ITT and PP population, in the HA (44.2% and 46.2%) compared to the P group (29.3% and 30.6%, for ITT and PP, respectively) at week 12 (*p* = 0.020 and 0.024, respectively), as displayed in Figure 3A,B.

Accordingly, the BMI reduction in PP population was significantly higher in HA compared to P groups (0.97 kg/m^2^ vs. 0.82 kg/m^2^, pU = 0.048). Absolute weight values at week 12 were not significantly different in the ITT analysis. In both groups, weight loss compared to baseline was significant: 2.89 kg (HA) and 2.49 kg (P). This difference (0.4 kg) was significant in PP (*p* = 0.046) with a trend in ITT population (0.3 kg; *p* = 0.10). No significant change was found in ITT population for either male (*p* = 0.12) or female (*p* = 0.28) subsets. A moderate, non-significant, increase of lean mass/fat mass ratio was found in both groups (Supplementary Table S1). 

**Figure 4:**
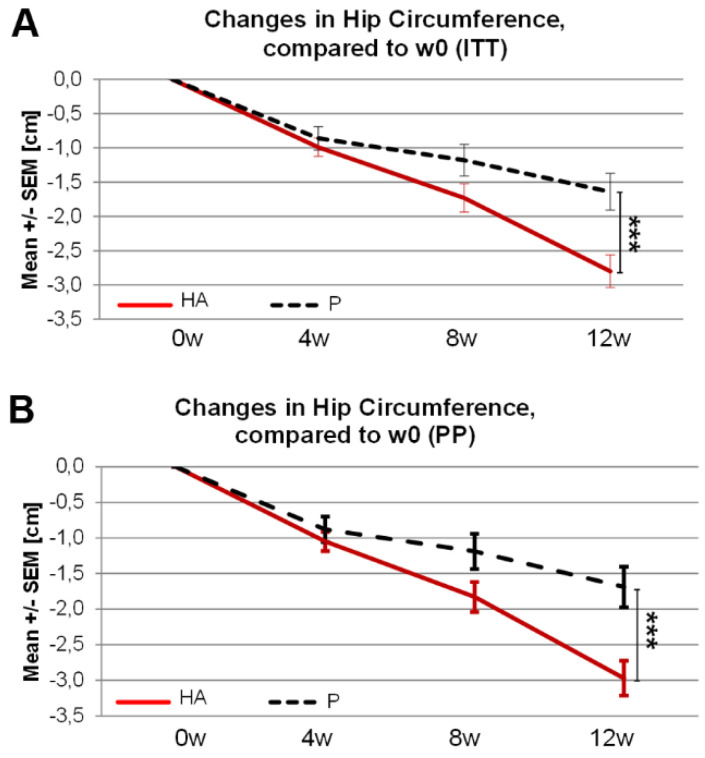
Changes in hip circumference following 12 weeks of HA supplementation in overweight subjects. Changes in Hip circumference following 12 weeks of HA supplementation compared to week 0 in ITT (**A**) and PP (**B**) population. (**A**,**B**) Mann-Whitney-U test (w12-w0)P. vs. (w12-w0)HA.*** p_U_ ≤ 0.001.

A significantly greater reduction of hip circumference at 12w was observed in the HA vs. P group in the ITT and PP population (Figure 4A,B). Waist circumference was not significantly changed in the ITT analysis while, in the PP analysis, a trend toward reduced waist circumference was observed in HA (−2.95 cm) compared to P (−2.76 cm) groups (pu = 0.10).

**Figure 5:**
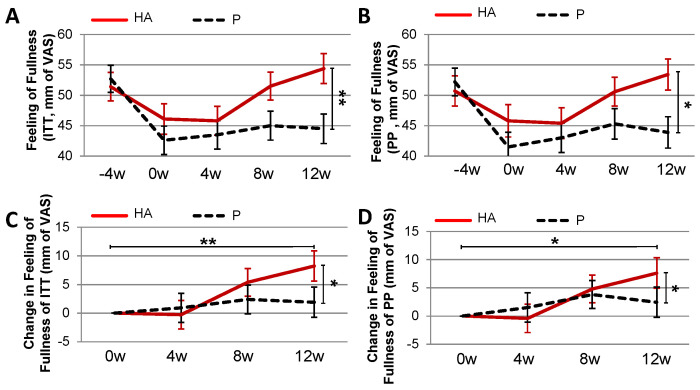
Feeling of fullness in overweight subject treated with HA under hypocaloric diet. Feeling of fullness in ITT (**A**) and PP (**B**) populations. Changes in the feeling of fullness over 12 weeks of HA supplementation in ITT (**C**) and PP (**D**) population. (**A**,**B**) Mann-Whitney-U test (w12)P. vs. (w12)HA.** pU ≤ 0.01.*pU ≤ 0.05. (**C**) Mann-Whitney-U test; (w12-w0)P. vs. (w12-w0)HA.* pU ≤ 0.05. Paired Wilcoxon test; HA(w0) vs. HA(w12).** pwi ≤ 0.01 (**D**) Mann-Whitney-U test; (w12-w0)P. vs. (w12-w0)HA.* pU ≤ 0.05. Paired Wilcoxon test; HA(w0) vs. HA(w12).* pwi ≤ 0.05.

The feeling of fullness at baseline was low in both groups (Figure 5). It increased significantly at 12 w in the HA (*p* = 0.01) versus P group (ITT and PP analysis, Figure 5A,B). The change in feeling of fullness from baseline to 12 w (Figure 5C,D) tended also (*p* = 0.085) to be higher in the HA group (8.24 mm Visual Analogue Scale; VAS) compared to P group (1.92 mm VAS). There were no differences between the groups for the feelings of satiety and craving (data not shown).

Gastrointestinal tolerance was good in both groups. Interestingly, a higher proportion of subjects in HA versus P groups reported a reduction of upper gastrointestinal (GI) symptoms at 12 w versus baseline (pHA = 0.003 and pP = 0.16, ITT analysis). Apart from this, no differences between groups were observed in changes of other GI symptoms. 

#### 3.2.3. Clinical and Biological Safety Evaluation 

No safety concern was observed both for clinical (data not shown) and biological safety parameters (Table 2). In ITT analysis, fasting glycemia was significantly lower at week 12 in the HA versus P group (pU = 0.027). There were no significant differences between groups regarding haemoglobin, haematocrit, erythrocytes, thrombocytes, leukocytes, ALAT, ASAT AP, gGT, bilirubin, creatinine, urea, uric acid, HbAc1, cholesterol and triglycerides. There were no difference in physical function domain of the IWQOL-LITE (pU = 0.637), systolic or diastolic blood pressure or pulse rates between the study groups, according to inclusion criteria, at the screening visit. Quality of life was not different between groups at 12w (ITT analysis) for the global IWQOL-LITE score and for any of the domains.

#### 3.2.4. Global Evaluation of Efficacy and Tolerability and Adverse Events (AE)

**Figure 6:**
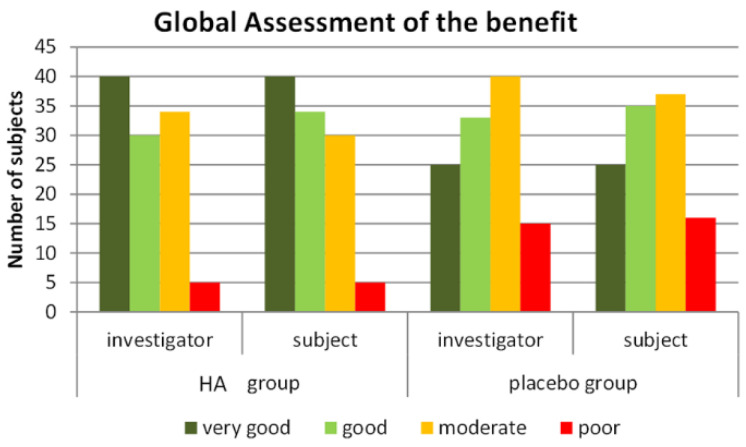
Global assessment of the treatment by the blinded subjects and investigators in the HA and P groups.

In the HA group, benefit of treatment (Figure 6) was rated as “very good” or “good” by 67.9% of subjects compared to 53.1% of subjects in the placebo group (pU = 0.019). The blinded investigators rated the benefit as “very good” or “good” for 64.2% of subjects in the HA group compared to 51.3% of subjects in the placebo group (pU = 0.035). Rating was “poor” by 5% of the HA subjects versus 14.2% of the P group. Tolerability was assessed by subjects and investigators as “very good/good” in 98.2% of cases in both groups (1.8% ratings were “moderate”).

Regarding AE, 43 out of 236 subjects (18.2%) reported a total of 55 AE: 25 in the HA group (21.2%) and 18 in the P group (15.3%, no statistical difference, pexF = 0.312). None of the AE was classified as “serious adverse event”. The causal relationship of AE to IP was classified as “unlikely” in all cases, excepted 2 AE classified as “not assessable”, one in each group. The intensity of AE was “mild” for 33 AE (22 subjects in the HA group, 11 subjects in the placebo group) and “moderate” in 22 AE (eight in the HA group and 14 in the P group). No AE was classified as “severe”.

## 4. Discussion

The present study investigated in overweight subjects the clinical efficacy of *H. alvei* HA4597^®^, a probiotic strain expressing ClpB, an α-MSH mimetic protein, and the main endpoint confirmed a higher rate of significant weight loss in subjects receiving HA in addition to hypocaloric diet. Increasing evidence has accumulated on the capacity of the gut microbiota to contribute to the regulation of body weight, body composition as well as host feeding behavior [6,21,22,23]. Accordingly, dietary interventions, including pre- and probiotics have been used to influence these parameters via modulation of gut microbiota composition in overweight and obese individuals, as well as in malnourished patients [24,25,26,27]. Several conventional probiotics have been proposed for obesity, including Lactobacillus (*L. casei*, *L. gasseri*, *L. plantarum*, *L. rhamnosus*) and Bifidobacterium (*B. infantis* and *B. longum*) species [11,28,29,30]. However, until now, the mechanisms by which these strains may reduce excess weight remain unclear and their clinical efficacy has not yet been demonstrated convincingly [11,28,29]. More specifically, none of these probiotics have been reported to stimulate satietogenic pathways. In contrast, in the present study, we show that the supplementation with H. alvei HA4597^®^ increased significantly the proportion of overweight subjects losing at least 3% and even 4% of baseline body weight while following a hypocaloric diet over 12 weeks. Recent expert guidelines underline that aiming to lose 3–5% of body weight is a meaningful objective for overweight patients, since this change has been associated with clinically significant improvements such as reduced blood glucose, reduction of type-2 diabetes and cardiovascular risks [2]. Thus, our main finding of a better rate of weight loss at the level of 3% (ITT) and even 4% should be considered as clinically relevant to reduce the risk of later complications [2]. In addition, it was associated with reduced hip circumference, and a slight yet significant reduction of blood glucose. In previous studies in overweight or obese patients with full dose treatment (120 mg tid) of orlistat, the increase of percentage of patients achieving 5% weight loss after one year ranged 18–24% [31]. Thus, our finding of a 32% increase of 3% weight loss responders over 12 weeks (primary endpoint) and 50% increase of 4% weight loss responders (post-hoc endpoint) competes well with results with orlistat. It is common finding with drugs targeting overweight and obesity to report responders and non-responders [3], which reflects the heterogeneity of the underlying mechanisms and the different mechanisms of action of the drugs, and a high “placebo effect”. 

In our study, the placebo effect was a combination of the dietetic received by both groups and the placebo itself; despite a high response rate in the P group (41.4% at the 3% level and 29.3% at the 4% level), we were able to detect a marked, significant, increase in response rate. This specific benefit of HA supplementation was observed in addition to the effect of the hypocaloric diet followed by subjects in both groups. This may be related to the increased feeling of fullness in the HA group, that is likely to have made it easier for subjects to follow the diet and led to a greater global satisfaction. Accordingly, Figure 6 indicates a marked difference of perception of efficacy between the HA and P groups, both for subjects and for physicians. Thus, three months of HA supplementation may help for developing durable and healthy dietary habits.

The observed improved rate of body weight reduction was most likely due to the effect of HA on eating behavior through the production of ClpB by HA. Indeed, the new-generation probiotic used in the present study has been technically developed to overproduce the ClpB protein and ultimately enhance the activation of peripheral and central satiating pathways through the activation of melanocortin receptor [15]. Indeed, the melanocortin system is a key regulator of energy metabolism via transmission of anorexigenic signals and also by enhancing energy expenditure and lipolytic effects [32]. Melanocortin receptors are also present in intestinal mucosa, i.e., directly accessible to gut bacteria-derived products such as ClpB [33]. Thus, although food intake estimated from patients’ diary did not allow to detect a significant difference, the achievement of the main endpoint of this double-blind study is likely to be related to the satietogenic effect of the probiotic supplementation. Nevertheless, we cannot exclude that a better reduction of body weight and hip circumference in the HA group may also involve some direct lipolytic and/or thermogenic effect of ClpB. It seems unlikely that such reduction was related to physical activity because it was asked to be maintained at a similar level in both groups.

The present clinical results are well in line with our previous demonstrations of the efficacy of HA supplementation in mouse models of obesity (HFD-fed and ob/ob hyperphagic mice). Indeed, obese mice supplemented with HA showed a significant reduction in body weight gain associated with reduced food intake as well as reduced fat mass gain [15,16]. Accordingly, in the present study, HA supplementation increased the feeling of fullness in subjects of the HA as compared to placebo-group. We did not observe a difference for the feeling of “satiety”; this may be related to the fact that “satiety” is less well defined and easy to identify for lay people than fullness. The effect on the feeling of fullness (i.e., enhancing satiation) is likely to have facilitated the compliance of the subjects to the hypocaloric diet limiting the risk of compensatory compulsive behavior, and it is in accordance with known effects of alpha-MSH and other melanocortin receptor agonists [34].

The rationale for supplementation of subjects with weight excess with ClpB-producing probiotic is further reinforced by the data showing a significant decrease of Enterobacterales ClpB gene richness in the fecal microbiota of obese patients [15]. An independent study also demonstrated that ClpB-like gene function in fecal microbiota correlated negatively with BMI and fat mass and that obese subjects displayed low prevalence of bacterial taxa expressing ClpB with alpha-MSH homology [35]. These recent findings are in agreement with an earlier observation of lower abundance of Enterobacteriaceae in obese subjects [36]. In contrast to obesity, bacterial ClpB production was increased in mice with the activity-based anorexia and in food-restricted rats [37,38]. Altogether, these findings suggest that a high abundance of bacteria expressing the ClpB gene such as HA4597^®^ in the intestinal microbiota is associated with an enhanced satiety and reduced body weight.

In addition to a better weight loss response, HA supplementation significantly improved the reduction of hip circumference compared to placebo, and tended to decrease the waist circumference. The effects on waist circumference were, however, expected to be modest since the overweight subjects included in the present study did not exhibit a clinical pattern of excess visceral fat with metabolic syndrome features, but rather a subcutaneous fat accumulation. Therefore a combination of HA with physical activity able to reduce visceral fat needs to be addressed in a different study design, including obese patients with a visceral fat phenotype associated with insulin resistance, diabetes and related increased cardiovascular risk [39,40]. In line with the type of fat distribution observed in the subjects of this study, metabolic parameters were within the normal range at screening visit, without criteria for metabolic syndrome. After 12w of HA supplementation, plasma levels of fasting glucose were significantly lower in the HA group, however still in the normal range. This interesting result is in line with our preclinical findings in obese rodents [15] and warrants further investigations in prediabetes patients to evaluate a metabolic benefit of HA4597^®^. Recently, a probiotic approach targeting insulin resistance has been developed to compensate the depletion of Akkermansia muciniphila in the microbiota of obese and overweight patients with insulin resistance [41]. In this pilot study, Depommier and colleagues observed that *A. muciniphila* supplementation over 3 months improved insulin sensitivity, insulinemia, total cholesterol as well as other metabolic markers including hepatic enzymes (γ-glutamyl transferase and aspartate-aminotransferase). However, no significant changes in anthropometric parameters were observed, with only a trend toward decreased body weight (*p* = 0.09), fat mass (*p* = 0.09) and hip circumference (*p* = 0.09) compared to placebo, while no difference in waist circumference was reported among groups [41]. It is tempting to speculate that a combination of H. alvei HA4597^®^, *A. muciniphila* and eventually other probiotics may provide beneficial additive or synergistic anti-obesity and metabolic effects in obese patients. 

Quality of life improved in both groups of the present study, without any differences between groups. Indeed, it has been previously reported that body weight loss was associated with improvement of quality of life in obese patients [42]. A longer duration of HA supplementation would be probably needed to result in a greater improvement of quality of life in treated than placebo subjects. Gastrointestinal tolerance was good and no specific adverse events have been reported by overweight subjects supplemented with HA. This underlines the commensal nature of HA and its wide safety margin [43,44]. The safety of our precision probiotic approach with H. alvei is worth to be underlined since many of the single or combined drugs proposed for the treatment of overweight or obesity have a poor tolerance profile [3,4]. Ex. appetite suppressing drugs sibutramine and rimonabant which targeted the central control of food intake have been withdrawn from the market, due to unacceptable serious side effects [3,31].

Due to technical constraints, a limitation of this study is the lack of data about intestinal satiety hormone production and fecal microbiota composition in subjects before and after HA supplementation. Such analysis would allow to see if the ClpB gene enrichment in the HA-supplemented subjects may correlate with the clinical efficacy of HA4597^®^ and to determine its impact on other beneficial gut bacteria. 

## 5. Conclusions

In conclusion, this study demonstrates the efficacy of HA on body weight loss associated with a reduction of hip circumference and a greater feeling of fullness in overweight subjects. To the best of our knowledge, this is the first clear demonstration of the efficacy of a probiotic strain on weight loss and satiation in a prospective randomized placebo-controlled study. Supplementation with HA4597^®^ represents an innovative and well-tolerated strategy to enhance the efficacy of dietary advice for the control of excess body weight; the “precision probiotic” HA4597^®^ [45] paves the way to the precision medicine and nutrition by identifying responders thanks to a gut microbial-based personalized approach. Our probiotic should be further evaluated in conditions of excess fat accumulation and related metabolic disorders, as it may offer a safe and economically affordable alternative to the drugs recently licensed for the treatment of obesity.

## Figures and Tables

**Table 1:** Baseline characteristics of study subjects.

Characteristics	Total	ITT HA group	P Group	*p*	Total	PP HA group	P Group	*p*
Frequency	230	113	117	-	212	104	108	-
Gender m/f	103/127	51/62	52/65	1.000	97/115	47/57	50/58	0.891
Body height [cm]	173.1 ± 10.1	173.4 ± 10.9	172.9 ± 9.4	0.986	173.2 ± 10.1	173.4 ± 10.9	173.1 ± 9.4	0.791
Body weight [kg]	83.8 ± 10.8	84.1 ± 11.5	83.4 ± 10.2	0.817	83.8 ± 10.9	84.1 ± 11.7	83.5 ± 10.2	0.931
BMI [kg/m²]	27.8 ± 1.4	27.9 ± 1.4	27.8 ± 1.4	0.909	27.8 ± 1.4	27.9 ± 1.4	27.8 ± 1.4	0.744
Body fat content [%]	32.3 ± 7.3	31.9 ± 7.4	32.6 ± 7.3	0.414	32.1 ± 7.3	31.9 ± 7.3	32.4 ± 7.3	0.582
Body fat mass [kg]	26.7 ± 5.6	26.4 ± 5.3	26.9 ± 5.8	0.563	26.5 ± 5.5	26.4 ± 5.3	26.7 ± 5.7	0.730
Fat free mass [kg]	57.2 ± 11.9	58.0 ± 12.7	56.5 ± 11.1	0.488	57.3 ± 12	57.9 ± 12.8	56.8 ± 11.2	0.660
Waist circumference [cm]	101.3 ± 9.1	101.0 ± 9.4	101.6 ± 8.9	0.656	101.1 ± 9.3	100.7 ± 9.7	101.4 ± 8.9	0.568
Hip circumference [cm]	106.2 ± 7.0	106.1 ± 6.7	106.2 ± 7.3	0.761	106.0 ± 7.0	106.1 ± 6.7	105.9 ± 7.3	0.547
Systolic blood pressure [mmHg]	126.6 ± 10.8	125.8 ± 11.0	127.4 ± 10.7	0.319	126.7 ± 11	126.1 ± 11.4	127.4 ± 10.6	0.519
Diastolic blood pressure [mmHg]	79.8 ± 6.2	79.9 ± 6.1	79.6 ± 6.2	0.629	79.7 ± 6.3	79.9 ± 6.3	79.5 ± 6.3	0.461
Pulse [bpm]	69.7 ± 7.8	70.4 ± 8.0	69.0 ± 7.6	0.313	69.5 ± 7.9	70.3 ± 8.2	68.7 ± 7.7	0.313

Intention to treat, ITT; Per protocol, PP; H alvei HA4597^®^, HA; P, Placebo; p, Exact Mann-Whitney U test *p* value; BMI, Body Mass Index.

**Table 2:** Laboratory parameters in ITT analysis.

Parameters [Unit]	−4w	HA Group w12	-4w	P Group w12	P_u_ W12 (HA v P)
Haemoglobin [mmol/L]	8.78 ± 0.82	8.75 ± 0.83	8.75 ± 0.75	0.82 ± 0.73	0.452
Haematocrit [L/L]	0.422 ± 0.036	0.424 ± 0.038	0.422 ± 0.035	0.426 ± 0.034	0.478
Erythrocytes [Tpt/L]	4.78 ± 0.44	4.77 ± 0.45	4.73 ± 0.42	4.68 ± 0.42	0.905
Thrombocytes [Gpt/L]	260.0 ± 58.1	265.9 ± 61.2	261.4 ± 48.1	266.8 ± 56.6	0.657
Leukocytes [GpT/L]	6.57 ± 1.84	6.60 ± 1.81	6.57 ± 1.92	6.57 ± 1.84	0.904
ALAT [µkat/L]	0.499 ± 0.292	0.468 ± 0.362	0.454 ± 0.223	0.408 ± 0.173	0.621
ASAT [µkat/L]	0.430 ± 0.116	0.439 ± 0.192	0.412 ± 0.138	0.416 ± 0.109	0.068
AP [µkat/L]	1.25 ± 0.343	1.27 ± 0.341	1.171 ± 0.314	1.170 ± 0.318	0.018
gGT [µkat/L]	0.406 ± 0.270	0.37 ± 0.22	0.397 ± 0.282	0.363 ± 0.261	0.479
Biluribin [µmol/L]	10.04 ± 5.2	10.4 ± 4.6	10.71 ± 5.76	12.00 ± 6.22	0.069
Creatinine [µmol/L]	74.5 ± 11.8	75.3 ± 12.6	74.9 ± 12.1	77.3 ± 11.8	0.181
Urea [mmol/L]	4.81 ± 1.3	4.69 ± 1.10	4.89 ± 1.40	4.84 ± 1.25	0.508
Uric acid [µmol/L]	311.4 ± 80.6	310.1 ± 83.2	317.4 ± 82.1	322.2 ± 81.8	0.368
Glucose [mmol/L]	5.46 ± 1.2	5.38 ± 0.3	5.49 ± 0.44	5.52 ± 0.53	0.027
HbA1c [%]	5.37 ± 0.29	5.38 ± 0.26	5.34 ± 0.24	5.36 ± 0.25	0.447
Cholesterol [mmol/L]	5.40 ± 1.16	5.19 ± 1.27	5.40 ± 1.08	5.28 ± 1.01	0.229
HDL- Cholesterol [mmol/L]	1.45 ± 0.38	1.43 ± 0.36	1.52 ± 0.34	1.49 ± 0.32	0.112
LDL-cholestetol [mmol/L]	3.53 ± 1.00	3.40 ± 1.09	3.53 ± 1.07	3.44 ± 0.92	0.423

## Data Availability

The data presented in this study are available from TargEDys upon request at glambert@targedys.com (accessed on 31 May 2021). The data are not publicly available due to confidentiality reasons.

## Supplementary Materials

The following are available online at https://www.mdpi.com/article/10.3390/nu13061902/s1.
