# Supplementary material for: The Probiotic Strain H. alvei HA4597® Improves Weight Loss in Overweight Subjects under Moderate Hypocaloric Diet: A Proof-of-Concept, Multicenter Randomized, Double-Blind Placebo-Controlled Study
Source: Nutrients. 2021 Jun 1;13(6):1902. doi: 10.3390/nu13061902 (PMC8227740; doi:10.3390/nu13061902)
Supplement: Supplementary file 1 [file nutrients-13-01902-s001.zip › nutrients-1214697-supplementary.pdf]

Table S1. Changes in Body composition assessments among groups

Characteristics	Total	ITT<br>HA group	P group	p	Total	PP<br>HA group	P group	p
Lean mass at 0w [Kg]	56.4±11.8	57.0±12.5	55.9±11.1	0.634	56.5±11.9	56.9±12.6	56.1±11.2	0.774
Lean mass at 12w [Kg]	56.0±11.6	56.6±12.5	55.3±10.7	0.636	56.0±11.7	56.5±12.6	55.5±10.8	0.749
Fat mass at 0w [kg]	26.1±5.6	25.9±5.2	26.3±5.9	0.615	26.0±5.5	25.8±5.2	26.1±5.8	0.772
Fat mass at 12w [Kg]	24.0±5.9	23.5±5.6	24.4±6.2	0.268	23.8±5.9	23.4±5.6	24.1±6.1	0.337
Lean/Fat mass ratio at 0w	2.31±0.86	2.35±0.89	2.27±0.82	0.482	2.32±0.85	2.35±0.88	2.30±0.83	0.606
Lean/ Fat mass ratio at 12w	2.54±1.00	2.60±1.02	2.47±0.97	0.291	2.56±1.00	2.62±1.03	2.51±0.98	0.377

Intention to treat, ITT; Per protocol, PP; H alvei HA4597®, HA; P, Placebo; p, Exact Mann-Whitney U test p value.
